# Benefits and Challenges of the Use of Two Novel vB_Efa29212_2e and vB_Efa29212_3e Bacteriophages in Biocontrol of the Root Canal *Enterococcus faecalis* Infections

**DOI:** 10.3390/jcm11216494

**Published:** 2022-11-01

**Authors:** Magdalena Moryl, Aleksandra Palatyńska-Ulatowska, Agnieszka Maszewska, Iwona Grzejdziak, Silvia Dias de Oliveira, Marieli Chitolina Pradebon, Liviu Steier, Antoni Różalski, Jose Antonio Poli de Figueiredo

**Affiliations:** 1 Department of Biology of Bacteria, Institute of Microbiology, Biotechnology and Immunology, Faculty of Biology and Environmental Protection, University of Lodz, 90-237 Lodz, Poland; 2 Department of Endodontics, Chair of Conservative Dentistry and Endodontics, Medical University of Lodz, 92-213 Lodz, Poland; 3 Laboratory of Immunology and Microbiology, School of Health and Life Sciences, Pontifical Catholic University of Rio Grande do Sul–PUCRS, Porto Alegre 90619-900, Brazil; 4 Department of Morphological Sciences, Federal University of Rio Grande do Sul – UFRGS, Porto Allegre 90010-150, Brazil; 5 Robert Schattner Center, School of Dental Medicine, University of Pennsylvania, Philadelphia, PA 19104, USA; 6 Federal University of Rio Grande do Sul—UFRGS, Porto Alegre 90035-003, Brazil; 7 Center for Transdisciplinary Research (CFTR), Saveetha Dental College, Saveetha Institute of Medical and Technical Sciences, Chennai 602105, Tamil Nadu, India

**Keywords:** bacterial drug resistance, bacteriophages, biofilm, endodontics, *Enterococcus faecalis*

## Abstract

Bacteriophage therapy has emerged as a strategy supplementing traditional disinfection protocols to fight biofilms. The aim of the study was to isolate the phages against *E. faecalis* and to characterize its biological features, morphology, and lytic activity in a formed biofilm model. Methods: *E. faecalis* ATCC 29212 strain was used for the trial. Two novel vB_Efa29212_2e and vB_Efa29212_3e virulent phages were isolated from urban wastewater and characterized. The *E. faecalis* biofilm was established in 15 bovine teeth for 21 days. Transmission (TEM) and scanning electron (SEM) microscopes with the colony-forming unit (CFU) counting were used for assessment. Results: Isolated phages differed in morphology. Taxonomy for vB_Efa29212_2e (Siphoviridae, Efquatovirus) and for vB_Efa29212_3e (Herelleviridae, Kochikohdavirus) was confirmed. Both phages were stable at a temperature range of 4–50 °C and showed a different tolerance to chemicals: 15% EDTA, 1–3% sodium hypochlorite, and chlorhexidine. SEM analysis showed distortion of bacteria cells after phage inoculation, which proved the lytic activity against *E. faecalis*. A 54.6% reduction in the *E. faecalis* biofilm confirmed bacteriophage efficacy against isolates in the ex vivo model. Conclusions: Results strongly support the concept that phage therapy has a real therapeutic potential for the prevention and treatment of *E. faecalis*-associated infections.

## 1. Introduction

Phages are the most common biological entities on Earth. They are simply called viruses destroying bacteria [1,2]. Bacteriophages consist of nucleic acid (DNA or RNA) and a protein shell called the capsid [3]. Only virulent phages, which always replicate via the lytic cycle, are suitable for phage therapy, as they enable safe and effective destruction of bacterial cells [4]. A virulent bacteriophage attaches to the receptors (protein, LPS, fimbriae, sugar molecule) on the surface of the host cell. Subsequently, it injects nucleic acid into the cytoplasm and, by the transcription of its own DNA, changes the host’s metabolism. Bacterial cells are lysed after immediate replication of the virion. As soon as the cell is destroyed, the phage progeny can find new hosts to infect. Fast and cheap methods of phage isolation, exponential increase in the number of bacteriophage particles after reaching the site of infection, and finally their high specificity are the features which make them a good therapeutic tool [5]. Bacterial resistance to the most used antibiotics is a matter of major concern. Evolution of the phages maintain their capability to destroy bacteria with a resistance to the treatment. Furthermore, bacteriophages may act synergistically with antibiotic treatment. Combined therapy can effectively prevent the rise of phage-resistant variants [6]. They also synthesize polysaccharide depolymerases, which destroy the biofilm [7]; thus, they are highly efficient in bacterial biofilm eradication, even in hard-to-reach tissues (long and tiny root canals) or spaces (e.g., medical equipment such as catheters).

### The Role of Enterococcus faecalis in a Root Canal Treatment (RCT) Failure

A thorough chemo-mechanical cleaning and shaping of root canals reduces the primary bacterial load by 95% [8], giving a suspected high success rate of the endodontic therapy. Unfortunately, the remaining viable bacteria together with possible changes in the microenvironment with time create favorable conditions for secondary or refractory endodontic infections [9] and may lead to the development of refractory inflammatory periapical lesions [8]. Regrowth of microorganisms in cases of insufficient root canal treatment may lead to recolonization of the endodontic space [10]. It can suppress the action of lymphocytes and potentially contributes to endodontic failure [11,12]. In chronic periapical periodontitis, there is a dynamic balance between the bacteria in the canal system and the host response in the surrounding tissues. When this balance is disturbed, exacerbation occurs spontaneously with severe symptoms due to the local production of pro-inflammatory and immune factors. The increased expression of various cytokines and growth factors promote angiogenesis in inflamed periradicular tissues [13]. The reasons for endodontic failure are a wide range of clinical factors such as quality and thoroughness of disinfection protocols, improper coronal seal, missed canals, and poor access to the biofilm due to anatomical constraints [14]. Depending on the amount of initial bacterial load left in the canal system, virulence of bacteria, and their resistance mechanisms, the development of secondary persistent infections may occur.

Numerous studies demonstrate that *E. faecalis* is the most frequently recovered species from teeth with persistent infections, resistant to conventional therapeutic procedures, persisting and multiplying in the root canal space [9,11,12,15,16]. In failed endodontic treatments, local changes in root canal milieu predispose the higher prevalence of *E. faecalis*. Xu et al. [17] showed that instrumentation and irrigation can alter the properties of the dentin surface and enhance the initial adhesion of *E. faecalis* to the root canal. Contrary to other species, *E. faecalis* can grow in the root canal niche under ecological stresses: low oxygen, alkaline pH, and nutritional deprivation [18,19]. It may maintain viability for twelve months without additional nutrients using fluid in periodontal ligaments and serum from dentin for nourishing. What is more, serum allows the bacteria to adhere to and invade the dentinal tubules. *E. faecalis* forms a biofilm in accessory canals, apical deltas, and isthmuses and is protected from antimicrobials by residual tissues, dentinal tissues, human serum, and dead cells [12,20]. In these cases, the eradication of *E. faecalis* from dentinal tubules by chemical agents, such as irrigants and intracanal dressings of calcium hydroxide or antibiotics, seems to be ineffective [21]. In biofilms, bacteria are embedded in self-produced polymeric substances, resist destruction, and become 1000 times more resistant to phagocytosis, antibodies, and antimicrobials than non-biofilm-producing organisms [22,23]. Cells from the deeper layers of the biofilm have restricted access to nutrients and oxygen, leading to considerably slower growth. This makes older biofilms more difficult to remove than younger biofilms [24]. Due to the high resistance of *E. faecalis* biofilms, many antibacterial methods are investigated based on both synthetic or natural agents, such as antibiotics, bacteriocins, or bacteriophages [25,26,27].

The isolation of new phage strains has become an important alternative in medicine due to the increasing antibiotic resistance of bacteria. Phages can be used as an interesting treatment option thanks to less bacterial resistance against this therapy and its relatively high specificity, which is less likely to impact the commensal flora. The advantages of phage therapy are the bacteriophage’s ability to multiply at the infection site, its efficacy against biofilms, and being a natural product, which is likely to be devoid of apparent toxicity [26,28]. 

The aim of the study was to isolate the phages against *E. faecalis*, characterize their morphology using transmission electron microscopy (TEM), and evaluate their biological features (the latent period and the burst size analysis). In the study, the obtained phages were examined in terms of their resistance/stability to environmental factors used during root canal treatments (RCT) such as sodium hypochlorite, EDTA, and chlorhexidine. The influence of temperature, pH, and UV light on the stability of the phages was also analyzed. The final objective of this in vitro trial was to observe the activity of the phages against the biofilm inoculated in the tubules of root canal dentin using a scanning electron microscope (SEM) and UV light fluorophore mapping.

## 2. Materials and Methods

### 2.1. E. faecalis Phage Isolation 

An *Enterococcus faecalis* ATCC® 29212TM strain was cultivated using brain heart infusion (BHI) (Difco Laboratories, Detroit, MI, USA) for 18–24 h at 37 °C, and it was used for all experiments. The bacteriophages were isolated from urban wastewater from the Group Sewage Treatment Plant in Lodz, Poland. The wastewater samples were centrifuged at 3600× *g* at 4 °C for 30 min, filtered through a paper filter (Schleicher and Carl Chull), and next through a sterile filter with a pore diameter of 0.2 μm (Sartorius, Goettingen, Germany). A total of 4 mL of purified wastewater mixed with an equal volume of 2× concentrated BHI and 0.5 mL of 18-hour *E. faecalis* culture on BHI were incubated for 24 h at 37 °C with shaking (150 rpm). Next, the culture was centrifuged at 3600× *g* at 4 °C for 30 min and was filtered through a sterile filter (0.2 μm). Double agar overlay plaque assay was used to detect and enumerate bacteriophages in the obtained filtrates [29]. Single plaques were cut out and transferred to tubes containing BHI medium supplemented with 50 µL of 18 h bacterial culture. After 24 h of incubation at 37 °C with shaking (150 rpm), the cultures were centrifuged and filtered as described above. The single-plaque excision procedure was repeated 5 times to obtain pure phage lysates that were stored at 4 °C [30].

### 2.2. Phage Morphology

A total of 1 mL of phages (titer 2 × 10^9^ PFU/mL) was centrifuged at 24,500× *g* for 3 h at 4 °C. Subsequently, the obtained pellet was washed twice with a 5% ammonium molybdate solution (Sigma-Aldrich, St. Louis, MO, USA), pH 6.0, and was suspended in 5% ammonium molybdate to the titer of 10^11^ PFU/mL. Next, a drop of the suspension was placed onto a formvar and carbon-coated copper grid (TED Pella, Inc., Redding, CA, USA) and was allowed to absorb the sample on the filter paper. The phages were stained with 2% uranyl acetate (Polysciences, Inc., Warrington, PA, USA) for 1 min and were observed under a transmission electron microscope (TEM), JEOL 1010 TEM, at 80 kV under the magnification of 60,000–100,000×. Phages were measured (15 virions of phage 2e and 18 virions of phage 3e) using the NIS-Elements D 5.11.01 software. The identification and classification of phages were conducted according to the guidelines of the International Committee on the Taxonomy of Viruses (ICTVs). Phages were classified using Ackermann’s viral specifications [31]. 

### 2.3. Phage DNA Isolation, Sequencing, and Bioinformatic Analysis

At the first stage of the experiment, the genomic phage DNA was isolated using a Phage DNA Isolation Kit (Norgen Biotek Corporation, Thorold, ON, Canada) as per the manufacturer’s instructions. The quality of the isolated DNA was checked by electrophoresis in agarose gel. 

The sequencing library was prepared by random fragmentation of the DNA sample followed by 5′ and 3′ adapter ligation. Sequencing was carried out at the Biobank Lab, University of Lodz (Poland), using an Illumina NextSeq sequencer with 2 × 150 bp paired-end readings. Next, bioinformatic analysis was performed. The general characterization of genome quality was performed using the Quast web application (http://cab.cc.spbu.ru/quast/, accessed on 22 November 2021). MultiPhate2 v.2.0.2 (https://github.com/carolzhou/multiPhATE2/, accessed on 22 November 2021) was used for ORF identification and characterization. The probable taxonomic classification was established based on a comparison with the nucleotide collection database using the nBLAST web version using the default megablast settings (https://blast.ncbi.nlm.nih.gov/, accessed on 23 November 2021). The platform PhageAI, Bacteriophage Life Cycle Recognition with Machine Learning and Natural Language Processing policy (https://phage.ai/, accessed on 5 September 2022), was used to define the phage’s life cycle [32].

### 2.4. Latency Period and the Burst Size

A total of 1 mL of *E. faecalis* culture was centrifuged at 12,000× *g* for 5 min at room temperature. The cells were washed in SM buffer, 50 mM Tris-HCl (Serva, Heidelberg, Germany), 100 mM NaCl (Eurochem, Tarnów, Poland), 0.01% gelatin (Sigma, St Louis, MO, USA), and 8 mM MgSO_4_•7H_2_O (Chempur, Piekary Slaskie, Poland), and the pellet was resuspended in 5 mL of SM buffer to obtain a bacterial density of 2 × 10^5^ CFU/mL. The number of bacteria was determined at the beginning of the experiment by assessing CFU/mL. The phages were added to the bacterial suspension at a phage-to-bacteria ratio of 1:10. The mixture was incubated for 5 min at 37 °C to adsorb the phages to the bacterial cells. This sample was centrifuged at 12,000× *g* for 5 min. The pellet was washed in the SM buffer to remove unadsorbed phages, was suspended in 20 mL BHI, and was incubated at 37 °C for 1.5 h. Every five min, the titer of phages was determined by the double agar layer method using BHI agar plates. The plates were incubated for 24 h at 37 °C, and the phage titer was calculated. The burst size was calculated using the formula: burst size = (B − A)/D, where B—the mean value of the titer from the plateau after the release of phages from the cell, A—the mean value of the titer from the plateau before the release of phages from the cell, and D—the number of infecting phages, the difference between the initial phage titer and the titer at time zero. The experiment was performed in triplicate. Phage titers were determined by plating appropriately diluted phage suspensions into at least three BHI agar plates.

### 2.5. Phage Sensitivity to Physicochemical Factors: pH, Temperature, UV Light, and Chemicals

The phage suspension diluted to a titer of about 10^6^ PFU/mL in BHI was exposed to physicochemical factors for 10, 30, and 60 min to determine its stability under various conditions. After incubation, the titer of phages was determined by the double agar layer method. 

The phage sensitivity to pH was assessed in BHI with different pHs (3, 4, 7, 9, 10 and 11) at room temperature. The pH value was adjusted with 1M HCl (Avator, Gliwice, Poland) or 2M NaOH (Avator, Gliwice, Poland), and the medium was sterilized by filtration (0.2 µm). Phages incubated in BHI with a pH of 7 served as positive controls.

To determine the stability of the phage titers at different temperatures, the phage suspensions were incubated at −20 °C, 4 °C, 37 °C, 50 °C, 60 °C, 70 °C, and 80 °C. Phages incubated at 4 °C (optimal condition for phage lysate storage) served as positive controls. 

Phages were exposed to a UV lamp emitting radiation with a wavelength of 254 nm at room temperature. Positive controls were phages incubated at room temperature and not exposed to UV radiation.

The sensitivity of phages to 2% chlorhexidine digluconate (CHX) (Gluxodent, Chema-Elektromet, Rzeszow, Poland), 3%, 2%, and 1% sodium hypochlorite NaOCl (Chempur, Piekary Slaskie, Poland), and 15% ethylenediamine acetic acid (EDTA (VDental, PFO Ventos-Farma, Bielawa, Poland) was examined. The irrigants were sterilized using filtration (0.2 µm). 

### 2.6. Biofilm Model Formation

Bovine incisors (*n* = 15) were stored in 1% sodium hypochlorite (ASPER, Indústria Química Ltda, São Caetano do Sul, Brazil) and were prepared for the trial [33]. Dental crowns and 1 mm of the apical region were sectioned to gain 15 mm long roots. Longitudinal buccolingual grooves were cut using a diamond bur (Dhpro, Rhadartrade, Paranaguá, Brazil). For the pulp removal, each root was rinsed with 2% sodium hypochlorite (ASPER, Indústria Química Ltda, São Caetano do Sul, Brazil) and instrumented up to the size #60 (Dentsply-Maillefer, Ballaigues, Switzerland). The roots were immersed under agitation in 17% EDTA (Farmashop, Porto Alegre, Brazil) for 5 min to remove the smear layer. Samples were randomly assigned to the test group (*n* = 9, including 2 teeth for fluorophore mapping), positive control (*n* = 3), and negative control group (*n* = 3).

Samples were fixed in polypropylene microtubes (Genuine Axygen Quality, Arizona, CA, USA) according to Gründling et al. [33]. After mounting, specimens were autoclaved (Kavo, Joinville, Brazil) at 121 °C for 15 min. A sterile paper cone was put inside a random sample to check the sterilization process. The cone was inoculated in a 0.85% sterile saline and homogenized. A total of 100 µL of this solution was spread on blood agar and incubated for 18 to 24 h at 37 °C. No contamination originating from the samplemind tooth was present. The specimen used for sterilization control was discarded. 

A 100 µL aliquot of the overnight culture of *E. faecalis* was inoculated into the tested tooth sample and maintained for 21 days at 37 °C, which is the optimal time for biofilm formation [34]. One third of the BHI was replaced every 48 h. Weekly, a part of the BHI was Gram stained, cultured in blood agar, and submitted to catalase and esculin tests to confirm the exclusive presence of *E. faecalis*. In the negative control group, *E. faecalis* was not inoculated.

A total of 1 mL of phage 3e (1 × 10^8^ PFU/mL) was added to each tested root. The bacteriophages were allowed to act for 48 h.

### 2.7. Biofilm Visualization (SEM, UV, CFU Counting)

Root fractures were completed using a #50 spatula (SS White, Rio de Janeiro, Brazil) to obtain two halves. After sectioning, 50% of the halves of the tested roots were used for SEM, and the other halves were used for colony-forming unit (CFU) counting. For biofilm disruption and subsequent CFU counting, each hemisectioned root was aseptically placed in a microtube with 1 mL of a 0.85% saline solution and sonicated in an ultrasonic water bath (Ultra Cleaner 1400A, Indaiatuba, Brazil) for 10 min. The saline-containing disrupted biofilm was diluted to 10^−3^. A total of 10 µL of each dilution was spotted on BHI agar in triplicate and incubated at 37 °C for 24 h. The number of CFUs remaining from bacteriophage-treated biofilms and their untreated counterparts was determined.

Specimens for SEM evaluation were immersed in a fixing solution (2.5% glutaraldehyde) for 7 days. Magnifications of 500× to 20,000× on a Phillips XL-30 scanning electron microscope (Eindhoven, Holland) were used. Samples were rinsed three times for 30 min, each time in a 0.2 mol/L phosphate buffer and distilled water at a 1:1 ratio. They were dehydrated by immersion in 30, 50, 70, 90, and 100% acetone and sputter-coated with gold for electron conduction. The medium third of the roots was chosen for analysis. Backscattering (BSE) was used for image capture. One observer blinded to the groups collected the images.

Samples were soaked with the fluorescent probe Invitrogen Qubit working solution (Thermo Fisher Scientific Inc., NYSE: TMO, Sao Paulo, Brazil) for fluorophore mapping. Visualization of *E. faecalis* biofilms under UV light (405 nm at a distance of 45 cm) with the use of REVEAL UV light loupes (Designs for Vision, New York, NY, USA) was performed. 

### 2.8. Statistical Analysis

Means and standard deviations (SD) were determined based on at least three independent repetitions of the experiments. Statistical analysis (Statistica 13.3, StatSoft Inc., Kraków, Poland) was carried out using the parametric Student’s t test when data were normally distributed as assessed by the Kolmogorov–Smirnov normality of residuals test. The nonparametric statistical test (Mann–Whitney U test) was used for data that were not normally distributed. Differences between the means of two groups were considered significant at *p* values < 0.01.

## 3. Results

### 3.1. Bacteriophage Morphology Assessment 

**Figure 1:**
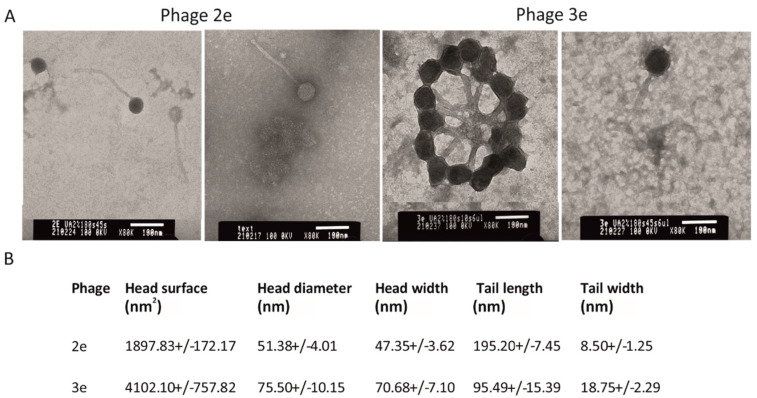
Electron micrograph of the 2e and 3e phages negatively stained with uracyl acetate (TEM). Magnification 80,000×, bars 100 nm; (**A**) head and tail dimensions are presented as mean ± SD. Statistical differences (Student’s *t* test) were observed between all tested morphological features of phages 2e and 3e (*p* < 0.01); (**B**) tail width values were measured at half length.

The evaluated bacteriophages differed significantly in morphology from each other. Phage 2e had a smaller head in comparison to phage 3e (1897.83 ± 172.17 nm^2^ and 4102.10 ± 757.82 nm^2^, respectively). The head shape of phage 2e was rounder, and its diameter was 51.38 ± 4.01 nm. The tail was 195.20 ± 7.45 nm in length, while its width in half of the length was 8.50 ± 1.25 nm. The capsid of the 3e phage had a diameter of 75.50 ± 10.15 nm. Its tail was almost two times shorter and wider than the 2e phage, with an average length and width of 95.49 ± 15.39 nm and 18.75 ± 2.29 nm, respectively (Figure 1). The 2e phage showed a typical morphology of Siphoviridae, while 3e represents the A1 morphological group (Myovirids). The features of 3e suggested it is a member of the Herelleviridae family.

### 3.2. Genomic Characterization of the Phages 

The phage vB_Efa29212_2e genome consisted of 41,351 nucleotides with a % G + C content of 34.83 and was assembled in a single contig. The phage encoded 75 putative open reading frames (ORFs) and zero tRNAs. Analysis of the 2e phage complete genome, based on the query cover of NCBI BLASTn, showed a high homology with Enterococcus phage SANTOR1 (KX284704.1), belonging to Efquatrovirus, Siphoviridae; (identity—95.48%; cover—88%; length—37,933).

The phage vB_Efa29212_3e genome was determined to be 141,162 bp in length with a 35.83% G + C content and was assembled in two contigs. The phage is predicted to encode 237 proteins and 9 tRNA genes. BLASTn analysis of the 3e genome revealed a high sequence similarity to Enterococcus phage 156 (LR031359.1), belonging to Kochikohdavirus, Brockvirinae, Herelleviridae (identity—98.58%; cover—88%; length—141,133 bp).

We applied a machine learning-based tool—PhageAI [32]—to predict if the phages are virulent or lysogenic. The obtained results allowed us to predict that both examined phages are virulent and follow a lytic life cycle (2e—96.11%; 3e—90.95%).

The genome sequences of the phages were deposited in the NCBI GenBank database (www.ncbi.nlm.nih.gov) under the accession numbers 2e: OP559177 and 3e: OP559178, OP559179.

### 3.3. Analysis of the Biological Features of the Phages

**Figure 2:**
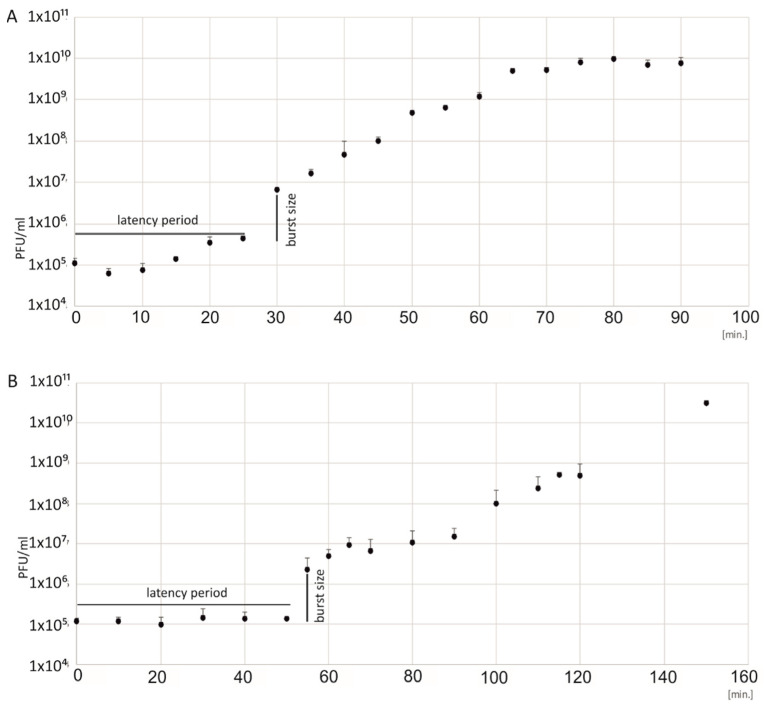
One-step growth curves of the 2e (**A**) and 3e (**B**) bacteriophages. The PFU/mL at different points in time are presented. Burst sizes, the number of phage particles per bacterial cell, were computed as 139 for phage 2e and 127 for phage 3e. The latency period was 25 min for the 2e phage and 55 min for the 3e phage. SD and mean value data are shown as log10 (PFU/mL).

The one-step growth curves of 2e and 3e on *E. faecalis* ATCC 29212 were determined. Based on the phage titer, the latency period and the average burst size values were obtained (Figure 2). Based on the curves, the latency period was calculated as 25 min for the 2e phage and 55 min for the 3e phage. The burst size was similar for both the 2e and 3e phages and was 138 and 127 viruses per bacterial cell, respectively. A large burst size accounts for their strong lytic activity against *E. faecalis*.

### 3.4. Analysis of Physiochemical Factors on the Phages

#### 3.4.1. Influence of pH 

**Figure 3:**
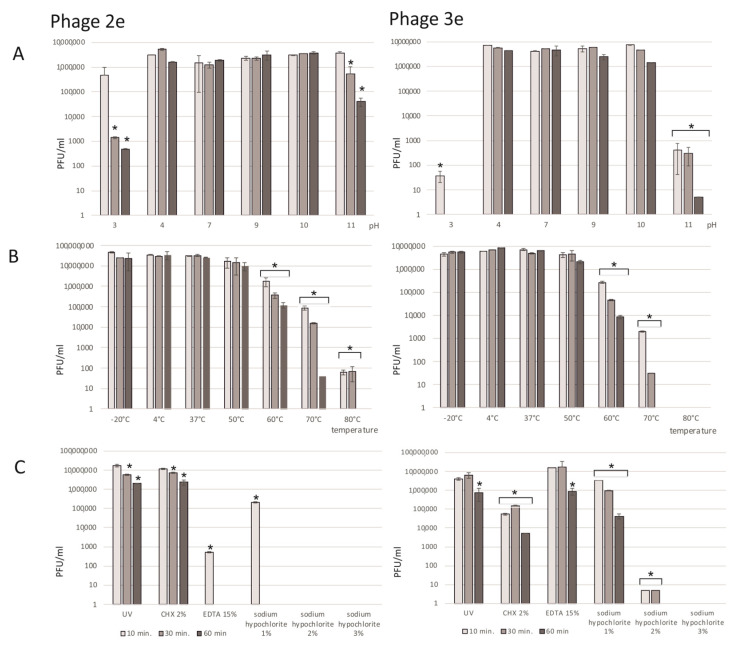
Effect of environmental factors on the 2e and 3e phages’ titers (PFU/mL). (**A**) pH: values are presented as log values of the ratio of phage titers in each treatment to that of the initial phage titer; (**B**) temperature: the phages are stable over a broad range of temperatures; (**C**) UV and chemical substances: chlorhexidine digluconate (CHX), 2%; ethylenediaminetetraacetic acid (EDTA) 15%; and sodium hypochlorite, 1–3%. Results are presented as the mean values ± SD from three independent experiments. Statistical significances were determined using the Mann–Whitney U test. Significant differences between the compared experimental variants and controls were marked by asterisk *p* < 0.01.

The assessment of the bacteriophages’ stability in acidic and basic environments was performed in pHs from 3 to 11 (Figure 3A). the evaluated phages were stable in pHs from 4 to 10; the average titers of 2e and 3e were similar to that obtained in a pH of 7. Incubation of the phages in environments with the border pH values of 3 and 11 resulted in extensive titer reductions. After 10 min of exposure to a pH of 3 and a pH of 11, the titer of phage 3e decreased to 37 PFU/mL and to 398 PFU/mL, respectively. Phage 2e was more stable in the extreme values of pH. After a longer (30 and 60 min) incubation in a pH of 3 and 11, a significant decrease in the titer of this phage was observed (down to 4.75 × 10^2^ in a pH of 3 and to 3.0 × 10^4^ in a pH of 11).

#### 3.4.2. Influence of Temperature

Both the 2e and 3e phages were tested in a wide range of temperatures (−20 °C, 4 °C, 37 °C, 50 °C, 60 °C, 70 °C, and 80 °C) over different time periods (Figure 3B). Phages incubated at 4 °C (optimal temperature for phage storage) were a positive control. The investigated phages were quite stable in a wide spectrum of temperatures. Short freezing of the samples for 10, 30, or 60 min did not influence the phage titers. The average titers at a temperature of −20 °C were 3.27 × 10^7^ PFU/mL for the 2e and 5.19 × 10^6^ PFU/mL for the 3e phage and were comparable to the values at the optimal temperature (4 °C). Phages were stable at up to 50°C, and their titers were similar to the positive control values. Incubation at 60 °C had a significant impact on the tested lysate titers, which were reduced by about 100–500 times. A strong destructive effect on the tested phages, especially for the 3e phage, was observed after heating at a temperature of 80 °C, where no phage particles were detected. Phage 2e was more resistant to heating, and the titer was 65 PFU/mL after incubation at 80 °C.

#### 3.4.3. Influence of UV Light on the Phages

UV light radiation for 10–60 min had a slight effect on the titer values of the evaluated phages (Figure 3C). A five-fold and nine-fold decrease in the titers of 3e and 2e, respectively, were observed after 60 min of UV exposure.

#### 3.4.4. Influence of Chemicals

Among all the investigated chemical agents, sodium hypochlorite influenced the phages the most (Figure 3C). After 10 min of incubation in a 3 and 2% solution of NaOCl, there were no phage molecules in the lysate. Phage 2e was more sensitive than 3e to 1% NaOCl, and the titer after 10 min of incubation was 2.25 × 10^5^ PFU/mL. It decreased to 0 after 30 min of incubation. Chlorhexidin digluconate and EDTA also had negative influences on the phage lysates. Depending on the time of action and the type of agent, a drop in titers from 2 to 500 times was observed. Phage 2e was less sensitive to CHX in comparison to phage 3e. However, phage 3e appeared to be more stable in the EDTA solution compared to phage 2e.

### 3.5. Activity of the Phages against the Biofilm in the Root Canal Dentin Walls 

**Figure 4:**
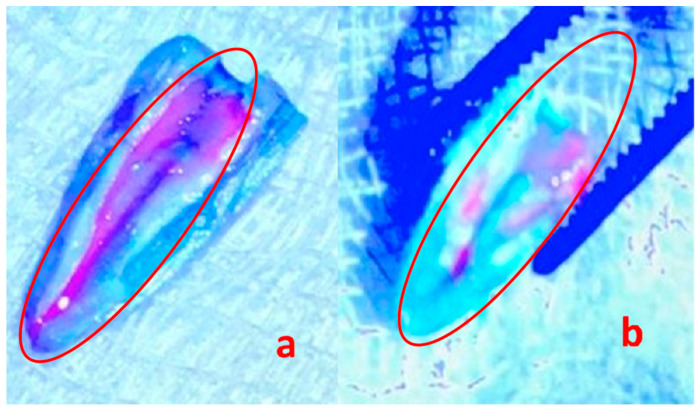
21-day-old *Enterococcus faecalis* biofilms on roots (**a**) untreated (positive control) and (**b**) treated with bacteriophage 3e visualized with the fluorescent probe Invitrogen Qubit observed under UV light. The red circle shows the purple endodontic biofilm (**a**) and the removal of a large part of the endodontic biofilm after 48 h of incubation with bacteriophages (**b**). Magnification 500×.

The UV light visual analysis of the biofilms in the test and control groups displayed the effect of phage 3e on the reduction in *E. faecalis* (Figure 4). From the untreated biofilms (positive control), an average of 1.4 × 10^7^ CFU/mL was obtained, while in the bacteriophage-treated biofilms an average of 6.36 × 10^6^ CFU/mL was found, resulting in a reduction of 54.6% of *E. faecalis* forming biofilms after treatment with phage 3e for 48 h.

**Figure 5:**
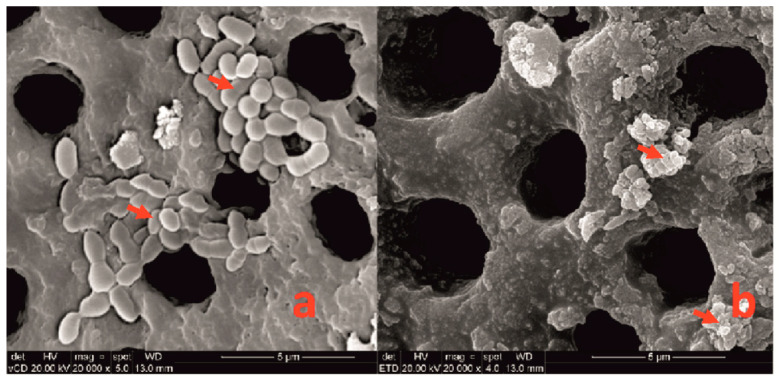
Scanning electron microscopy of *Enterococcus faecalis* 21-day-old biofilms on roots (**a**) untreated (positive control) and (**b**) treated for 48 h with bacteriophage 3e. Note that the structure of the bacteria is totally modified or destroyed by the phages (arrows). Magnification 20,000×.

SEM demonstrated a clear disruption of the bacterial cells after the phage therapy (Figure 5). Their different shape and complete loss of structure confirm the lytic activity of phage 3e. All test samples displayed the same profile, both under UV light and SEM analyses.

## 4. Discussion

Finding virulent phages specific for *E. faecalis* with potential therapeutic efficacy in endodontics is a subject of current research [27,35]. The phages, in contrary to other disinfectants and antibiotics, could combat multi-drug-resistant bacteria, penetrating and propagating through the biofilm that forms in the root canal space, and as a result eradicate the biofilm. Phage therapy is beneficial, safe for patients, preserves the composition of the microbiota, and induces minor side effects. In such therapies, phages with high host susceptibility, high adsorption rates, short latency periods, and large burst sizes should be selected [36].

In this study, we isolated two bacteriophages, vB_Efa29212_2e and vB_Efa29212_3e, specific against *E. faecalis*, belonging to various families: Siphoviridae and Herelleviridae. Genome analysis has revealed that both of the studied phages are virulent. They showed specific biological features which make them good candidates for phage therapy. Phages 2e and 3e remained active in unfavorable physical conditions, i.e., relatively low and high pHs and temperatures, as well as UV radiation. Another advantage of the phages was that during the lysis of the host cell, a large number of daughter virions were released, which subsequently were able to infect cells of the pathogen. A short period of time from the host cell infection to virion release was indicated especially for the 2e phage. A phage with a short latency period multiplies fast and in a short period of time which causes lysis of the bacterial cells. Furthermore, bacteriophage 3e was highly potent against *E. faecalis* biofilms. 

Endodontic procedures reduce most of the root canal biofilm, but some resistant bacteria survive and lead to treatment failure [6,37]. A standard protocol for cleaning and shaping of the canal system includes instrumentation with files and the use of irrigation agents (dissolving the rest of the pulp, removal of the smear layer) with ultrasonic activation (increase in temperature) and intracanal dressings (rise in pH). The influence of irrigation solutions commonly used in RCTx for the cleaning and shaping of canal systems on the viability of bacteriophages was not yet discussed in the literature, which is a benefit of the presented study. When designing an effective therapy, it should be taken into account that *E. faecalis* can survive and grow in harsh environments such as high pHs and a temperature of 45 °C [38]. The described 2e and 3e phages are stable in a wide range of temperatures (–20 up to 50 °C) and pH values (4–10). It makes them potentially ideal to use in endodontic treatments. However, the thesis stated by Lee et al. [39] about the combinatory treatment with alkaline disinfectants such as sodium hypochlorite seems not to be confirmed. Solutions with 5.25–6% sodium hypochlorite are considered as the main, very effective irrigants against intracanal microbiota. Together with mechanical canal debridement, they have the ability to eradicate bacterial biofilms. Thus, their role in potential synergistic phage therapy is clinically relevant. Nevertheless, based on the presented results, not only the alkaline environment, but also the concentration of the agent matters. NaOCl has a strong activity against phages. Only for the concentration of 1%, the titer of both phages slightly diminished and did not cause elimination of the virions. A big drop in the titer of the 3e phage to 8 PFU/mL and a total loss of the 2e phage in the presence of 2% NaOCl was observed. Concentrations of sodium hypochlorite above 3% kills the bacteriophages. Therefore, the use of NaOCl in a standard 5.25% concentration is not recommended in phage therapy and can implicate problems from a clinical point of view. On the contrary, the use of EDTA or chlorhexidine seem to be more suitable. EDTA as well as CHX may be combined with phage therapy in both acidic and alkaline environments and in higher temperatures. Further studies on the synergistic activity of irrigants are needed; however, it appears that specified chemicals may serve as a conjoint protocol, not influencing phage activity.

Phage-based dental therapies mainly should be targeting bacterial biofilms. Various research models can be used to study the antibiofilm properties of new bacteriophages, including those that most closely reflect in vivo conditions [34]. Phee et al. [40] identified two phages, JBD4 and JBD44a, and studied their antibiofilm activity against the Pseudomonas aeruginosa PA14 biofilm formed on microplates and on the extracted human tooth model. Interestingly, biofilm reduction was significant only in the microplate model. In the present study, it turned out that in the hard-accessible root canal space application, the 3e virion was beneficial. We observed a high efficiency of phage 3e in the eradication of *E. faecalis* biofilms. In the initial stage of the study, while the biofilm was inoculated on polystyrene plates, we observed a higher potential of the 3e phage than the 2e phage against *E. faecalis* biofilms. That is why, in the first round, the 3e one was taken for the trial. Despite the administration of a single dose of the lysate for 48 h, over a 50% reduction in the number of viable bacterial cells in the biofilm was observed. A long 21-day biofilm cultivation period was used to obtain its mature, fully developed structure. SEM analysis revealed the destruction of the treated biofilm which, compared to the intact one, looked like clumps of distributed bacteria. In terms of clinical relevancy, we applied one dose of the phages administered for 48 h. It was used in the experiment in this way, to maintain the phages in between visits, mimicking clinical situations. These are the pilot studies that constitute the starting point for research into effective phage dosing to obtain successful biofilm eradication in the future. 

There are only a few studies on *E. faecalis* phages used in endodontic treatments, which are consistent with the results described in the presented study. Lee et al. [39] described a HEf13 phage with a broad activity against several clinical isolates of *E. faecalis*. The virion, belonging to the Siphoviridae family, is characterized as a lytic one with similar features and a potential therapeutic efficacy. In the Paisano et al. trial [41], the teeth were infected with *E. faecalis* and assessed in terms of the effect of the phages on the viability of the bacteria. Phage lysates also led to a substantial reduction in bacteria viability. Another EFDG1 *E. faecalis* phage isolated from sewage water has shown efficiency in eliminating a greater portion of a 2-week-old *E. faecalis* biofilm of ~100 µm thickness, causing a five-log reduction in biomass compared to the untreated biofilms [27]. 

There are some factors important to improve the efficacy of phage therapy: phage concentration, routes of administration, dosing frequency, and duration of therapy [42]. All factors mentioned above should be taken into consideration to develop a fully effective therapy. Single-dose administration may be insufficient for biofilm eradication. To maximize concentrations of phages at infection sites, the Members of the Antibacterial Resistance Leadership Group (ARLG) Phage Taskforce recommended the administration of a high-titer preparation and repeated dosing for extended durations [36]. Onsea et al. [43] set up the efficient treatment protocols of *E. faecalis* infection in which phages were administered for 10 days, three times per day. The selection of a well-fitted dosage, frequency, and duration of 3e virion lysate administration would lead to the complete elimination of *E. faecalis* biofilms from the root canal. For a good clinical outcome, these aspects must be considered and thoroughly evaluated. Future research will allow for better standardization, accuracy, and application of phage therapy in clinical conditions. Nevertheless, we speculate that in antibiotic-resistant bacterial infections of endodontic origin in vivo, these novel virulent phages are promising and are a justified direction of future research. We believe that it should be applied in challenging cases of persistent infections, when routine treatment does not achieve a good outcome. Due to phage susceptibility to sodium hypochlorite, we recommend using the phages after a conventional disinfection protocol.

The limitation of our study, which needs to be considered, is testing only the phages against mono-species *E. faecalis* biofilms. Thus, the direction of further research shall include studies on the activity of a single or combined bacteriophage against both planktonic forms and multi-species biofilms of different strains of bacteria. In addition, the comparison of the investigated bacteriophages with common endodontic disinfectants such as CHX or Ca(OH)_2_ should be indicated.

## 5. Conclusions

Phages can actively penetrate and disturb biofilms in nature. The virions evaluated in the study destroyed *E. faecalis* biofilms in root canal system. If *E. faecalis* biofilms are formed within the depths of endodontic spaces, they can be used to obtain improved treatments, followed by regular endodontic procedures. Their biological features (large burst size and short latency period), together with a small sensitivity to physical and chemical factors, make these entities good candidates for effective therapy, which may be of extreme potential help. 

## Data Availability

The genome sequences of the phages were deposited in the NCBI GenBank database under the accession numbers 2e: OP559177 and 3e: OP559178, OP559179 (www.ncbi.nlm.nih.gov).
